# Inequalities in the disease burden in Scotland: an area level analysis

**DOI:** 10.1093/eurpub/ckac129.401

**Published:** 2022-10-25

**Authors:** I Grant, E Fletcher, G McCartney, M Thrower, G Wyper, D Stockton

**Affiliations:** 1 Data Driven Innovation Directorate, Public Health Scotland, Edinburgh, UK; 2 College of Social Sciences, University of Glasgow, Glasgow, UK; 3 Place and Wellbeing Directorate, Public Health Scotland, Glasgow, UK; 4 Clinical and Protecting Health Directorate, Public Health Scotland, Glasgow, UK

## Abstract

In the context of increasing demand for evidence-based policy, attempts to address or mitigate the effects of disadvantage have been usefully informed by comprehensive indices of multiple deprivation. These indices combine indicators on a range of dimensions of deprivation to classify neighborhoods or localities. Through combining information on fatal and non-fatal health loss, burden of disease studies allow planners and policy-makers to have a better understanding of the contribution of different diseases and injuries to the total burden of disease. These estimates can be augmented through studies, stratified by investigating inequalities in the burden of disease due to area-based deprivation. Doing so, helps contribute to discussions about where prevention and service activity should be focused to address health inequalities. The Scottish Burden of Disease study uses the Scottish Index of Multiple Deprivation (SIMD) as means to report on of the extent of inequality in the burden of disease in Scotland between people living in the areas of greatest, and of least, multiple deprivation. The SIMD quantifies deprivation based on data zones, a geographical unit comparable to a postcode. Using pooled and weighted data from seven domains (employment, income, crime, housing, health, education and geographic access), each data zone is given a composite rank out of 6,505 data zones. The composite rank was then converted to a decile, with 1 assigned to the 10% most deprived data zones and 10 to the 10% least deprived. In this presentation we will show the key steps involved in undertaking an area-based analysis of health inequalities in the burden of disease in Scotland using results from the Scottish Burden of Disease 2019 study, and from our monitoring of COVID-19 disability-adjusted life years.

